# Mango Consumption Was Associated with Higher Nutrient Intake and Diet Quality in Women of Childbearing Age and Older Adults

**DOI:** 10.3390/nu16020303

**Published:** 2024-01-19

**Authors:** Kristin Fulgoni, Victor L. Fulgoni

**Affiliations:** Nutrition Impact, LLC., Battle Creek, MI 49014, USA; fulgonik@gmail.com

**Keywords:** NHANES, women of childbearing age, older adults, mango, nutrient intake, diet quality, fruit

## Abstract

Nutritional equity has been recognized as a priority in nutrition research, as reiterated by the new life-stage approach utilized by the Dietary Guidelines for Americans. Two of the life stages focused on are pregnant and lactating females as well as older adults, due to their unique nutritional needs and unique chronic health conditions. Both groups have low intakes of several nutrients, including those of public health concern, and suboptimal diet quality, underlining the importance to improve their dietary intakes. Incorporating fruit into their diets could help alleviate these gaps. Thus, the objective of the current study is to expand upon our current knowledge of the benefits of fruit within these two subgroups that DGA guidelines focus on and determine nutrient and food group intake as well as diet quality in women of childbearing age (WCA; as a proxy for pregnant and/or lactating females, *n* = 16,774) and older adult (*n* = 18,784) mango consumers compared with non-consumers, using NHANES 1988–1994 and 1999–2018. Both WCA and older adult mango consumers had greater than 20% higher intakes of fiber and vitamin C and WCA consumers had upwards of 10% higher folate, magnesium, and potassium intakes compared with non-consumers. Both groups of consumers had lower intake of saturated fat and older adults had lower intakes of protein and vitamin B12. Diet quality was 16 and 13% higher in WCA and older adult consumers compared with non-consumers, respectively. Additionally, mango consumers had lower beef, poultry, fish, and solid fat intakes and WCA consumers had higher whole grain intakes compared with non-consumers. This study suggests incorporating mango into the diet could increase select nutrient intake as well as diet quality in specific life stages of adult Americans.

## 1. Introduction

Nutritional equity is currently at the forefront of nutrition research, shifting the focus to ensuring individuals of all genders, ethnicities, economic statuses, and life stages have access to healthy food and adequate nutrient intake [1]. As part of this focus, the Dietary Guidelines for Americans (DGA) introduced a life-stage approach for the first time in the 2020–2025 cycle, tailoring food guidance to children younger than 2 years, pregnant and lactating females, as well as older adults [2]. This life-stage approach allows adjustments to dietary pattern recommendations for subpopulations in response to unique nutrient and food group needs. 

One of the reasons the DGA has focused on pregnant and/or lactating females (PLFs) is due to their unique health concerns. Pregnant women are at risk of several health conditions including gestational diabetes, preeclampsia, hypertension, and excessive gestational weight gain (GWG) [3]. Developing these conditions during this life stage poses a health safety risk to both the mother and baby, as well as to their long-term health [3]. Adhering to a diet composed primarily of nutrient-dense foods and consuming a high-quality diet is imperative for this population, since diet quality has been associated with a lower risk of several common health conditions including excessive GWG, hypertensive disorders, and preeclampsia [3,4,5,6]. While PLFs tend to have 15–17% higher diet quality scores as measured by USDA’s Healthy Eating Index 2015 (HEI-2015) compared with their counterparts, there is still considerable room for improvement [2]. In addition to suboptimal diet quality, PLFs also have inadequate intake of several nutrients of public health concern as well as other nutrients important for a healthy developing baby. The DGA encourages PLFs to increase consumption of iron, folate, calcium, magnesium, protein, fiber, potassium, and vitamin D, since current intakes among women 19–50 years of age are low with greater than 20% of the population below the estimated average requirement (EAR) for calcium, magnesium, and vitamin D. Additionally, less than 30% of the population had intakes greater than the adequate intake for potassium [7]. Increasing fruit consumption in this population could boost intake of some of these nutrients in addition to improving diet quality. 

A second life-stage group of DGA focus was older adults, defined as individuals 60 years and older, due to their susceptibility to chronic health conditions including heart disease, cancers, and bone/muscle diseases [2]. Additionally, energy requirements for this age group are lower than their younger counterparts but nutrient recommendations are similar or higher, making nutrient-dense food choices of even greater importance [2]. Despite this, large percentages of the older adult population fall below the EAR for numerous nutrients, with greater than 25% of the 71 years and older population below the EAR for calcium, magnesium, zinc, and vitamins A, C, D, and E [7]. Additionally, disparities in nutrient intake are present between genders in this population, with greater than 20% of women falling below the EAR for folate and vitamin B6 [7]. Similar to PLFs, the diet quality (HEI-2015 scores) of older adults are 7–13% higher than younger adults, but they continue to fall short of fruit intake recommendations [2]. 

Increasing diet quality and nutrient intake in PLFs and adults 60+ y can be achieved, at least partially, by encouraging further fruit consumption. The DGA recommends consuming between 1.5 and 2 cups of fruit daily for PLFs and adults aged 60 years and older [2,3] due to its associations with lower risk of several health outcomes including all-cause mortality, cardiovascular disease, type 2 diabetes, and adverse bone health [2,3]. Mangos, in particular, are high in several nutrients of public health concern, with one cup containing 2.6 g of fiber, 18.2 mg of calcium, 16.5 mg of magnesium, 277 mg of potassium, 71 µg of folate, and 60.1 mg of vitamin C at less than 100 calories [8]. Numerous studies have reported on the benefit of incorporating individual types of fruit in the diet such as citrus juices and mango to improving diet quality and nutrient intake, but less information is available on the benefit of these fruits within the PLF and older adult populations [9,10]. Additionally, a myriad of studies in the past decades have focused on nutrient intake and diet quality in various subgroups, including adolescents, children, and adults of differing age ranges, but the knowledge gap relating to PLFs and older adults is vast and including these subgroups in nutrition studies is of importance. Therefore, the objective of the current study was to utilize numerous cycles of National Health and Nutrition Examination Survey (NHANES) data to develop a large sample size and examine nutrient intake, diet quality, and food group intake of mango consumers compared with non-consumers in two of the life stages focused on in recent DGA guidelines: women of childbearing age (WCA) and older adults. It was hypothesized that mango consumers of both subgroups would have higher diet quality as well as higher intake of important nutrients compared with non-consumers, in alignment with previous studies on more generalized subgroups such as children and adults.

## 2. Materials and Methods

### 2.1. Data Sources and Subjects

NHANES is a government-run survey designed to continually measure nutrition and health in the USA and is nationally representative of the non-institutionalized population. The current study utilized the dietary component of NHANES, What We Eat in America (WWEIA), which consists of two 24 h dietary recalls utilizing the automated multiple pass method [11]. All NHANES subjects completed the first 24 h dietary recall in person, and the majority of subjects completed the second 24 h recall administered by telephone. Informed consent was obtained from all subjects in the survey and details on NHANES and its dietary recall methods are available on their website [12,13].

NHANES III (1988–1994) and NHANES cycles 1999–2018 were utilized in the current study with a focus on two specific life stages. One of the life-stage groups the DGA 2020–2025 focused on is PLF, which we interpreted as WCA to garner a much larger sample size. As such, women aged 15–44 y who were not currently pregnant or lactating were included in the study, with a sample size of 16,774 after exclusions for unreliable dietary recalls. A second focus of the DGA 2020–2025 was the older adult population, therefore the second study population examined consisted of adults 60 years and older (genders combined) with reliable dietary recalls, with a sample size of 18,784. 

### 2.2. Consumer Status, Nutrient and Food Group Intake, Diet Quality Measure

Mango consumers were defined as subjects who reported the raw mango food code (63129010) in either their 1st or 2nd dietary recall. Nutrient intake was obtained from day 1 and day 2 total nutrient intake files. Usual intake was determined using the two-part (frequency and amount) National Cancer Institute method utilizing both 24 h recalls [14]. Food group intake utilized WWEIA food categories (beef, poultry, seafood; beef, poultry, seafood, organ and cured meat, seafood, eggs, soy, nuts, and seeds; refined grain; whole grain; solid fats; dairy; fruits; grain; vegetables). Diet quality was measured using the Healthy Eating Index 2020 (an update to the HEI-2015 recently released by USDA) which measures dietary adherence to 2020 DGA recommendations [15]. The maximum HEI-2020 score is 100 points, consisting of 13 subcomponents: total vegetables, greens and beans, total fruit, whole fruit, whole grains, dairy, total protein foods, seafood and plant protein, fatty acid ratio, sodium, refined grain, saturated fat, and added sugars. Higher points are awarded for greater intake of foods the DGA recommends to consume (i.e., total fruit, total vegetables), and for lower intake of foods recommended to be limited (added sugars, refined grains, sodium, and saturated fat). Components total fruits, whole fruits, total vegetables, greens and beans, total protein foods, and seafood and plant protein each have a maximum score of 5, whereas all other components’ maximum score is 10 points.

### 2.3. Statistical Analysis

Data were adjusted for the complex sample design of NHANES and analyzed with SAS 9.4 (SAS Institute, Cary, NC, USA). Regression analyses (PROC SURVEYREG) were used to determine statistical differences between mango consumers and non-consumers. Tertiles of usual mango intake were developed for WCA and older adult consumers. Data were adjusted for covariates age, gender, ethnicity, physical activity level (sedentary, moderate, vigorous), poverty income ratio, weight status (normal, overweight, obese), current smoking status (never, current, previous), and energy intake (other than for energy). Statistical significance was set at *p* < 0.05.

## 3. Results

WCA who consumed mango had a mean intake of 90.1 ± 0.41 g with tertiles ranging from <85.0 to >94.9 g (Table 1). Older adult mango consumers had a mean intake of 91.3 ± 0.54 g with tertiles ranging from <83.2 to >99.4 g (Table 1). Male consumers had approximately 3.8 g higher intake compared with females.

### 3.1. Women of Childbearing Age

#### 3.1.1. Nutrient Intake

WCA who were mango consumers had higher daily intake of carbohydrates (21.0 ± 5.10 g), dietary fiber (4.32 ± 0.75 g), folate (DFE; 124 ± 44.8 µg), magnesium (40.5 ± 10.2 mg), potassium (243 ± 66.1 mg), total folate (91.4 ± 26.7 µg), total sugars (9.09 ± 4.56 g), and vitamins C (54.1 ± 12.6 mg) and E (2.30 ± 1.04 mg) compared with non-consumers (Table 2). Consumers also had lower daily intake of added sugars (−3.07 ± 1.02 tsp eq), total fat (−6.76 ± 1.90 g), total monounsaturated fatty acids (MUFA; −3.27 ± 0.70 g), and total saturated fatty acids (−2.70 ± 1.0 g) compared with non-consumers.

#### 3.1.2. Diet Quality

WCA mango consumers had a higher overall HEI-2020 scores (7.72 ± 1.45 points; pts) as well as higher total fruit (1.72 ± 0.20 pts), whole fruit (2.09 ± 0.18 pts), whole grains (0.81 ± 0.34 pts), sodium (0.70 ± 0.32 pts; corresponding to lower sodium intake), saturated fat (0.87 ± 0.37 pts; corresponding to lower saturated fat intake), and added sugar (1.08 ± 0.34 pts; corresponding to lower added sugar intake) component scores compared with non-consumers (Table 3).

#### 3.1.3. Food Group Intake

WCA mango consumers had higher intakes of total fruits (1.08 ± 0.15 cup eq), total grain (0.73 ± 0.32 oz eq), and whole grain (0.33 ± 0.15 oz eq) compared with non-consumers (Table 4). Mango consumers also had lower intakes of beef, poultry, and seafood (−0.74 ± 0.28 oz eq) and solid fats (−5.19 ± 1.77 g) compared with non-consumers.

### 3.2. Older Adults

#### 3.2.1. Nutrient Intake

Older adult mango consumers had higher intakes of carbohydrates (12.0 ± 5.86 g), dietary fiber (3.95 ± 1.18 g), and vitamin C (36.8 ± 8.53 mg) compared with non-consumers (Table 5). Mango consumers also had lower intakes of cholesterol (−33.6 ± 14.9 mg), niacin (−1.85 ± 0.77 mg), phosphorus (−59.2 ± 26.8 mg), protein (−6.91 ± 1.57 g), riboflavin (−0.13 ± 0.05 mg), total SFA (−2.25 ± 0.75 g), and vitamin B12 (−1.13 ± 0.24 µg) compared with non-consumers.

#### 3.2.2. Diet Quality

Mango consumers 60 years and older had a higher total HEI-2020 score (7.12 ± 1.47 pts) and higher component scores for greens and beans (0.59 ± 0.26 pts), total fruit (1.11 ± 0.17 pts), whole fruit (1.38 ± 0.15 pts), and fatty acid ratio (1.19 ± 0.35 pts) compared with non-consumers (Table 6).

#### 3.2.3. Food Group Intake

Older adult mango consumers had higher intake of total fruits (0.71 ± 0.12 cup eq) and lower intakes of beef, poultry, and seafood (−0.74 ± 0.23 oz eq), solid fats (−4.94 ± 1.44 pts), and total protein foods (meat, poultry, organ meat, cured meat, seafood, eggs, soy, nuts, and seeds; −0.67 ± 0.24 oz eq) compared with non-consumers (Table 7).

## 4. Discussion

The current study showed intakes of important nutrients, including several nutrients of public health concern, were higher in mango consumers in two specific life stages, WCA and older adults. Additionally, mango consumers had higher diet quality scores, with WCA and older adults having 16 and 13% higher HEI-2020 scores, respectively, compared with non-consumers. Food group intakes differed between mango consumers and non-consumers, with consumers in both subgroups having lower intakes of beef, poultry, fish, and solid fats in addition to higher whole grain intake for WCA consumers. This study suggests incorporating mango into the diet could be beneficial to nutrient intake as well as diet quality in specific life stages of adult Americans.

Due to the unique nutritional needs of PLFs including increased protein, iron, magnesium, folate, and potassium recommendations, the DGA has focused on this life stage during the 2020–2025 cycle. The DGA recommends PLFs to increase their intake of these nutrients among others, because they are currently under-consumed by greater than 10% of the pregnant population, with magnesium, iron, and vitamins D and E inadequacy being greater than 30% within the pregnant population [3,16]. The current study showed WCA who consumed mangos had higher intake of several of these nutrients, including folate, magnesium, fiber, potassium, and vitamin E, suggesting incorporating mangos into the diets of PLFs may help alleviate inadequacies of important nutrients. In addition to increased nutrient needs, the DGA also encourages WCA to increase their diet quality prior to becoming pregnant, during pregnancy, and throughout lactation [2]. While pregnant and lactating females tend to have higher diet quality scores compared with their counterparts, there is still considerable room for improvement [2]. A previous study reported pregnant women had relatively high HEI-2015 scores for fruits and whole grains whereas they had lower scores for dairy and protein components as well as foods recommended to limit [17]. In contrast, the current study found WCA who consumed mangos had higher scores for fruit components as well as sodium, saturated fat, and added sugar components, suggesting that including mango in the diet may further increase the diet quality of PLFs compared with their non-consumer counterparts. Regardless of mango consumer status, WCA fell greater than 25% below the maximum component score for fruit, underlining the need for increased fruit consumption in this population. While the benefits of fruits are well known in this population, numerous barriers exist for PLFs to consume a healthy diet, including finances, time constraints, physical pregnancy symptoms, and the quality of nutritional guidance provided by their care providers [18]. Ensuring all pregnant and/or lactating women have access to the same quality of care and nutritional education could help to alleviate a barrier to healthy eating during this important life stage.

Another reason the DGA has focused on pregnant and lactating females this cycle is due to this group’s unique health concerns including gestational diabetes, preeclampsia, hypertension, and excessive GWG. The DGAC, in addition to several previous studies, have found that a dietary pattern higher in fruit as well as other nutrient-dense foods can help reduce the risk of these conditions [3,4,5,6]. Fiber from fruit, in particular, has been shown to have a beneficial effect on gestational diabetes [5] and fruit and/or vegetable intake in addition to magnesium and calcium intake reduced the risk of hypertensive disorders or preeclampsia in pregnant women [4]. The present study found WCA who consumed mangos had higher total fruit intake as well as higher magnesium and fiber intake, suggesting that including mango in their diet may reduce the risk of these conditions. Findings on GWG are less consistent, with the DGAC reporting limited evidence suggesting dietary patterns with higher levels of nutrient-dense foods including fruits, vegetables, nuts, legumes, etc. carry a lower risk of excessive GWG [3]. More consistent findings are available for relationships between GWG and diet in women with obesity pre-pregnancy. Fruit and vegetable intake in this population protects against excessive GWG, but the findings were inconsistent in the total population [6]. It is possible that unique nutrients and nutrient matrices within individual fruits are associated with this protective effect of fruits since certain fruits have shown positive associations with common health conditions in PLFs, including apples [19]. Further research into individual fruits, including mango, and their association with the unique health concerns of PLFs is warranted to identify foods to incorporate into their diet and to ascertain whether causal relationships exist between individual fruit intake and nutrient intake and diet quality. Since this life stage is prone to taste sensitivity and food aversion [18], gaining knowledge on the benefits of various fruits would help give PLFs several options that meet their taste preferences while still gaining the benefits of fruit.

An important finding of the study is the association between mango consumption, higher intakes of healthy food groups and nutrients, and lower intake of food groups to limit. Of particular interest is the lower intake of added sugars associated with mango consumers in WCA. It is possible that mango consumers are utilizing fruit, and specifically mango, as a source of sweetness in replacement of energy-dense foods with high added sugar content. Additionally, it is possible that lower intake of added sugar during pregnancy could decrease future intake in the child, based on taste preferences as well as parental modeling of healthy eating practices.

The new DGA life-stage approach also focuses on older adults during the 2020–2025 cycle. Nutrition in older adults is of concern due to their decreased calorie requirements and similar or higher nutrient recommendations, making nutrient-dense food selections imperative in this population [2]. While older adults tend to have a slightly higher diet quality than their younger counterparts [2], the majority of the older adult population consumes more calories from added sugars and saturated fat than recommended, in addition to consuming higher than recommended levels of sodium [2]. This further accentuates the need for significant improvements in their diet quality and an increase in nutrient-dense food intake. Additionally, increasing diet quality in older adults could help ward off common chronic diseases observed in this population, including heart disease, cancers, and bone and muscle diseases, which represent an approximate total of 24% of disability-adjusted life years in adults 50 years and older [20]. Previous studies focusing on diet quality in older adults have reported positive associations between diet quality and health conditions, with a reported 12–28% decrease in all-cause, CVD, and cancer mortality risk in older adults in addition to positive relationships between HEI scores and a myriad of health conditions including diabetes, hypertension, CVD, cognitive decline, and sarcopenia [21,22,23,24]. One way to increase diet quality in older adults is to further encourage consumption of fruits since their intake is currently below DGA recommendations [2]. The current study and previous studies on other individual fruits suggest incorporating fruit into the diets of older adults would help to increase their diet quality as well as boost their immune system and decrease their risk of overweight and obesity, which commonly occurs in this population [9,25].

An interesting finding of this study was the lower intakes of protein and associated nutrients including vitamin B12, niacin, and riboflavin in older adult mango consumers compared with non-consumers. They also had higher component scores for greens and beans corresponding to higher intake as well as lower intake of total protein foods and beef, poultry, and seafood. Following these results, we performed a post hoc analysis on the older adult population to determine whether an association between vegetarian or vegan diets and mango consumption existed. Individuals who followed a vegetarian and/or vegan diet were identified two ways; self-identified and as subjects with zero meat consumption. In older adults, a higher percentage of mango consumers reported zero meat intake among combined genders and in analyses of females only (Supplementary Table S1). Similar findings were reported for the total US adult population, with mango consumers having higher seafood and plant protein HEI component scores [9]. In conjunction, these results suggest adult mango consumers are more likely to eat a vegetarian/vegan diet, which is more concerning in older adults who are more likely to under-consume protein and vitamin B12 and are at a heightened risk of malnutrition [2]. A recent review reported oral nutrition supplements and protein-fortified foods are common forms of protein supplementation in care facilities, but compliance and effectiveness have been inconsistent [26]. Determining whether these supplementations could be useful for all older adults and identifying pathways to incorporate more nutrient-dense foods high in protein and other pertinent nutrients could help balance out the current reduced intake in the total older adult population and among mango consumers.

This study has several strengths, including the usage of several cycles of NHANES, a large nationally representative survey, allowing generalization of the results. Additionally, a significant strength of this study was the focus on two life-stage populations recognized by the DGA; PLFs as represented by WCA and older adults. Studying these subpopulations helps to fill important knowledge gaps of the impact of consuming fruits, including mangos, in these understudied populations. Finally, the significant number of cycles utilized allowed a larger sample size of mango consumers in the studied subpopulations. On the other hand, this study has several limitations. NHANES is an observational study and therefore causal relationships cannot be assessed, and NHANES is dependent on dietary recalls, which have been subject to misreporting [27]. While WCA are not equivalent to PLFs, this proxy was utilized to expand the limited sample size of PLFs in NHANES. Additionally, a small percentage of the population consume mango, making it possible the study indirectly selected people with healthier diets possibly resulting in higher nutrient intakes and diet quality measures. The current study did not include dietary supplements as a covariate although they are common in the studied populations. Future research could focus on examining the nutrient intake benefits of mango and individual fruits independent of dietary supplement usage in PLC and older adults. Finally, the analyses were not stratified by demographic variables, which could be an important future study focus since a previous study found differences in mango consumer status based on ethnicity [10].

## 5. Conclusions

In conclusion, both WCA and older adults who consumed mangos had higher intakes of nutrients important in those life stages, in addition to higher diet quality and diet quality components. Identifying individual fruits that help to ward off common health conditions in WCA could give multiple options for this subpopulation who are prone to food aversions. Additionally, older adults who consumed mangos had lower intake of protein, vitamin B12, riboflavin, and niacin, as well as lower intakes of protein foods. Identifying ways to improve the protein intake of older adults while increasing fruit intake could be helpful to decrease any potential risk of malnutrition while preventing chronic health conditions.

## Figures and Tables

**Table 1:** Usual Intake ^1^ of Mangos by Consumers ^2^ (g/day) in women of childbearing age (15–44 y) and older adults (60+ y), NHANES 1988–1994, 1999–2018.

Population	Gender	*n*	Mean (SE)	Tertile 1	Tertile 2	Tertile 3
Women of Childbearing Age	Female	262	90.1 (0.41)	<85.0	85.0–94.9	>94.9
Older adults	All	272	91.3 (0.54)	<83.2	83.2–99.4	>99.4
	Male	116	93.7 (0.66)	<81.3	81.3–103	>103
	Female	156	89.9 (0.62)	<83.4	83.4–99.2	>99.2

Data source: NHANES 1988–1994, 1999–2018; female subjects aged 15–44 years (*n* = 16,774) and adults aged 60 years and older (*n* = 18,784) who were mango consumers with complete and reliable 24 h dietary recalls. y: years of age; ^1^ usual intake was determined with the National Cancer Institute two-part method using both 24 h recalls. ^2^ Mango consumers were defined as subjects who reported raw mango on either day 1 or day 2 24 h recalls.

**Table 2:** Nutrient intake ^1^ of mango consumers ^2^ and non-consumers in women of childbearing age (15–44 y), NHANES 1988–1994, 1999–2018.

Nutrient	All (*n* = 16,774)	Consumers (*n* = 213)	Non-Consumers (*n* = 16,561)	Consumer vs. Non-Consumer	
	Mean (SE)	Mean (SE)	Mean (SE)	Beta ^3^ (SE)	*p* ^4^
Added sugars (tsp eq)	18.45 (0.21)	15.41 (1.01)	18.48 (0.19)	−3.07 (1.02)	**0.0030**
Calcium (mg)	823.1 (6.39)	893.6 (61.2)	822.4 (5.11)	71.24 (60.9)	0.2436
Carbohydrate (g)	238.0 (1.21)	258.8 (5.00)	237.8 (0.76)	21.04 (5.10)	**0.0001**
Cholesterol (mg)	237.4 (2.18)	215.5 (17.1)	237.6 (2.02)	−22.14 (17.3)	0.2017
Choline (mg) ^5^	268.9 (2.32)	278.3 (13.4)	268.7 (1.87)	9.59 (13.7)	0.4870
Dietary fiber (g)	13.98 (0.13)	18.26 (0.75)	13.93 (0.10)	4.32 (0.75)	**<0.0001**
Folate, DFE (µg) ^6^	471.8 (5.20)	593.8 (44.3)	470.2 (4.52)	123.6 (44.8)	**0.0066**
Iron (mg)	12.95 (0.10)	13.85 (0.62)	12.94 (0.08)	0.90 (0.63)	0.1503
Magnesium (mg)	252.2 (1.90)	292.3 (10.6)	251.8 (1.37)	40.55 (10.2)	**0.0001**
Niacin (mg)	21.00 (0.14)	20.47 (0.84)	21.00 (0.12)	−0.53 (0.84)	0.5273
Phosphorus (mg)	1168 (6.68)	1166 (32.2)	1168 (4.27)	−1.28 (32.1)	0.9682
Potassium (mg)	2265 (14.9)	2506 (67.4)	2263 (10.9)	242.9 (66.1)	**0.0003**
Protein (g)	69.71 (0.38)	68.28 (2.19)	69.72 (0.27)	−1.44 (2.20)	0.5121
Riboflavin (Vitamin B2) (mg)	1.78 (0.01)	1.78 (0.07)	1.78 (0.01)	−0.001 (0.07)	0.9838
Sodium (mg)	3144 (18.2)	3031 (98.2)	3145 (12.8)	−113.9 (98.5)	0.2489
Thiamin (Vitamin B1) (mg)	1.41 (0.01)	1.48 (0.04)	1.41 (0.01)	0.07 (0.04)	0.1065
Total fat (g)	72.94 (0.43)	66.25 (1.89)	73.01 (0.26)	−6.76 (1.90)	**0.0005**
Total folate (µg) ^6^	349.7 (3.52)	439.9 (26.3)	348.5 (2.94)	91.42 (26.7)	**0.0008**
Total MUFA (g)	26.29 (0.16)	23.06 (0.70)	26.32 (0.10)	−3.27 (0.70)	**<0.0001**
Total PUFA (g)	16.29 (0.14)	15.49 (0.69)	16.30 (0.10)	−0.80 (0.68)	0.2410
Total SFA (g)	24.14 (0.15)	21.47 (0.99)	24.17 (0.11)	−2.70 (1.00)	**0.0073**
Total sugars (g)	113.6 (0.87)	122.6 (4.47)	113.5 (0.76)	9.09 (4.56)	**0.0472**
Vitamin A (RE)	622.1 (8.94)	688.2 (61.6)	621.4 (8.38)	66.73 (62.6)	0.2874
Vitamin B12 (µg)	4.15 (0.06)	3.81 (0.42)	4.15 (0.06)	−0.34 (0.40)	0.3973
Vitamin B6 (mg)	1.67 (0.01)	1.87 (0.13)	1.67 (0.01)	0.20 (0.13)	0.1289
Vitamin C (mg)	78.53 (1.17)	132.1 (12.6)	77.97 (1.05)	54.12 (12.6)	**<0.0001**
Vitamin D (D2 + D3) (µg)	3.90 (0.06)	4.32 (0.45)	3.89 (0.05)	0.42 (0.45)	0.3441
Vitamin E (ATE) (mg)	7.60 (0.09)	9.87 (1.03)	7.57 (0.08)	2.30 (1.04)	**0.0285**
Vitamin K (µg) ^6^	97.68 (2.73)	113.4 (15.9)	97.48 (2.58)	15.96 (15.5)	0.3049
Zinc (mg)	9.76 (0.07)	9.51 (0.39)	9.77 (0.06)	−0.26 (0.39)	0.5076

Data source: NHANES 1988–1994, 1999–2018; female subjects aged 15–44 years (*n* = 16,774) with complete and reliable 24 h dietary recalls. ^1^ Data were adjusted for age, gender, ethnicity, physical activity level, poverty income ratio, weight status, current smoking status, and energy intake (except for energy). ^2^ Mango consumers were defined as subjects who reported raw mango on either day 1 or day 2 24 h recalls. ^3^ Beta is a regression coefficient indicating differences between consumer and non-consumer. ^4^ *p* values are for difference between consumer and non-consumer intake. ^5^ Choline intake was only available for NHANES 2005–2018 cycles. Choline sample sizes: all (*n* = 8816); consumers (*n* = 156); non-consumers (*n* = 8660). ^6^ Folate, DFE, total folate, vitamin K intakes were only available in 2003–2018 cycles. Sample sizes: all (*n* = 12,601); consumers (*n* = 199), non-consumers (*n* = 12,402). Bold values designate significant differences at *p* < 0.05. y: years of age; SE: standard error, MUFA: monounsaturated fatty acids; PUFA: polyunsaturated fatty acids; SFA: saturated fatty acids.

**Table 3:** Healthy Eating Index 2020 ^1^ (HEI-2020) of mango consumers ^2^ and non-consumers among women of childbearing age (15–44 y), NHANES 1988–1994, 1999–2018.

HEI-2020 Component	All (*n* = 16,774)	Consumers (*n* = 213)	Non-Consumers (*n* = 16,561)	Consumer vs. Non-Consumer	
	Mean (SE)	Mean (SE)	Mean (SE)	Beta ^3^ (SE)	*p* ^4^
Component 1—total vegetables	3.01 (0.02)	3.02 (0.17)	3.01 (0.02)	0.01 (0.18)	0.9528
Component 2—greens and beans	1.37 (0.02)	1.74 (0.24)	1.37 (0.02)	0.37 (0.24)	0.1287
Component 3—total fruit	1.82 (0.03)	3.52 (0.20)	1.80 (0.03)	1.72 (0.20)	**<0.0001**
Component 4—whole fruit	1.66 (0.03)	3.72 (0.18)	1.63 (0.03)	2.09 (0.18)	**<0.0001**
Component 5—whole grains	2.02 (0.04)	2.82 (0.34)	2.01 (0.04)	0.81 (0.34)	**0.0166**
Component 6—dairy	5.19 (0.05)	5.15 (0.34)	5.20 (0.04)	−0.05 (0.34)	0.8861
Component 7—total protein foods	3.96 (0.02)	3.80 (0.17)	3.96 (0.02)	−0.16 (0.17)	0.3501
Component 8—seafood and plant protein	2.02 (0.03)	2.32 (0.25)	2.01 (0.03)	0.31 (0.25)	0.2192
Component 9—fatty acid ratio	4.81 (0.05)	5.25 (0.40)	4.81 (0.05)	0.44 (0.40)	0.2645
Component 10—sodium	4.45 (0.05)	5.14 (0.31)	4.45 (0.05)	0.70 (0.32)	**0.0300**
Component 11—refined grain	5.89 (0.04)	5.42 (0.39)	5.89 (0.04)	−0.48 (0.39)	0.2247
Component 12—saturated fat	5.89 (0.04)	6.76 (0.37)	5.88 (0.04)	0.87 (0.37)	**0.0202**
Component 13—added sugar	5.81 (0.06)	6.88 (0.34)	5.80 (0.05)	1.08 (0.34)	**0.0017**
HEI-2020 total score	47.89 (0.22)	55.54 (1.47)	47.81 (0.19)	7.72 (1.45)	**<0.0001**

Data source: NHANES 1988–1994, 1999–2018; female subjects aged 15–44 (*n* = 16,774) with complete and reliable 24-h dietary recalls. ^1^ Data were adjusted for age, gender, ethnicity, physical activity level, poverty income ratio, weight status, and current smoking status. ^2^ Mango consumers were defined as subjects who reported raw mango on either day 1 or day 2 24 h recalls. ^3^ Beta is a regression coefficient indicating differences between consumer and non-consumer. ^4^ *p* values are for difference between consumer and non-consumer intake. Bold values designate significant differences at *p* < 0.05. y: years of age; SE: standard error.

**Table 4:** Food group intake ^1^ of mango consumers ^2^ and non-consumers among women of childbearing age (15–44 y), NHANES 1988–1994, 1999–2018.

Food Group	All (*n* = 16,774)	Consumers (*n* = 213)	Non-Consumers (*n* = 16,561)	Consumer vs. Non-Consumer	
	Mean (SE)	Mean (SE)	Mean (SE)	Beta ^3^ (SE)	*p* ^4^
Beef, poultry, seafood (oz eq)	3.92 (0.04)	3.19 (0.28)	3.93 (0.04)	−0.74 (0.28)	**0.0098**
Refined grain (oz eq)	5.29 (0.04)	5.69 (0.33)	5.29 (0.03)	0.40 (0.33)	0.2297
Solid fats (g)	34.05 (0.23)	28.91 (1.78)	34.11 (0.19)	−5.19 (1.77)	**0.0037**
Total dairy (cup eq)	1.47 (0.02)	1.46 (0.11)	1.47 (0.01)	−0.01 (0.11)	0.9232
Total fruits (cup eq)	0.76 (0.02)	1.84 (0.15)	0.75 (0.01)	1.08 (0.15)	**<0.0001**
Total grain (oz eq)	5.91 (0.04)	6.63 (0.31)	5.90 (0.03)	0.73 (0.32)	**0.0217**
Total mpf, organ, cured, seafood, eggs, soy, nuts, seeds (oz eq)	4.90 (0.05)	4.40 (0.32)	4.91 (0.04)	−0.51 (0.33)	0.1233
Total vegetables excluding legumes (cup eq)	1.42 (0.02)	1.43 (0.12)	1.42 (0.01)	0.01 (0.12)	0.9187
Whole grain (oz eq)	0.62 (0.02)	0.94 (0.15)	0.62 (0.01)	0.33 (0.15)	**0.0283**

Data source: NHANES 1988–1994, 1999–2018; female subjects 15–44 years of age (*n* = 16,774) with complete and reliable 24 h dietary recalls. ^1^ Data were adjusted for age, gender, ethnicity, physical activity level, poverty income ratio, weight status, current smoking status, and energy intake. ^2^ Mango consumers were defined as subjects who reported raw mango on either day 1 or day 2 24 h recalls. ^3^ Beta is a regression coefficient indicating differences between consumer and non-consumer. ^4^ *p* values are for difference between consumer and non-consumer intake. Bold values designate significant differences at *p* < 0.05. y: years of age; SE: standard error; mpf: meat and poultry foods.

**Table 5:** Nutrient intake ^1^ of mango consumers ^2^ and non-consumers among older adults (60+ y), NHANES 1988–1994, 1999–2018, gender combined data.

Nutrient	All (*n* = 18,784)	Consumers (*n* = 225)	Non-Consumers (*n* = 18,559)	Consumer vs. Non-Consumer	
	Mean (SE)	Mean (SE)	Mean (SE)	Beta ^3^ (SE)	*p* ^4^
Added sugars (tsp eq)	13.25 (0.12)	12.04 (0.96)	13.26 (0.10)	−1.22 (0.97)	0.2091
Calcium (mg)	819.4 (6.79)	818.3 (32.2)	819.4 (4.72)	−1.07 (32.4)	0.9738
Carbohydrate (g)	222.1 (1.21)	234.0 (5.93)	222.0 (0.56)	12.05 (5.86)	**0.0409**
Cholesterol (mg)	255.6 (2.32)	222.3 (14.8)	255.9 (2.11)	−33.64 (14.9)	**0.0254**
Choline (mg) ^5^	309.3 (2.54)	293.7 (9.50)	309.5 (2.04)	−15.81 (9.50)	0.0991
Dietary fiber (g)	16.43 (0.13)	20.34 (1.18)	16.39 (0.10)	3.95 (1.18)	**0.0010**
Folate, DFE (µg) ^6^	499.3 (4.91)	508.2 (32.3)	499.2 (4.25)	9.03 (32.2)	0.7795
Iron (mg)	14.37 (0.11)	14.10 (0.95)	14.38 (0.08)	−0.28 (0.94)	0.7672
Magnesium (mg)	279.3 (1.81)	293.8 (10.8)	279.1 (1.20)	14.64 (10.9)	0.1792
Niacin (mg)	21.77 (0.17)	19.94 (0.76)	21.79 (0.14)	−1.85 (0.77)	**0.0177**
Phosphorus (mg)	1200 (7.66)	1142 (26.4)	1201 (3.99)	−59.22 (26.8)	**0.0280**
Potassium (mg)	2637 (14.7)	2764 (77.3)	2636 (9.70)	127.9 (77.6)	0.1009
Protein (g)	71.25 (0.44)	64.41 (1.55)	71.31 (0.26)	−6.91 (1.57)	**<0.0001**
Riboflavin (Vitamin B2) (mg)	2.00 (0.01)	1.87 (0.05)	2.00 (0.01)	−0.13 (0.05)	**0.0138**
Sodium (mg)	3083 (18.2)	2924 (97.9)	3084 (11.5)	−160.7 (98.4)	0.1041
Thiamin (Vitamin B1) (mg)	1.52 (0.01)	1.47 (0.05)	1.52 (0.01)	−0.05 (0.05)	0.3549
Total fat (g)	71.12 (0.45)	67.93 (2.11)	71.15 (0.21)	−3.22 (2.10)	0.1266
Total folate (µg) ^6^	375.4 (3.43)	400.2 (19.6)	375.1 (2.87)	25.12 (19.8)	0.2070
Total MUFA (g)	25.76 (0.17)	24.43 (0.95)	25.77 (0.10)	−1.34 (0.95)	0.1611
Total PUFA (g)	16.05 (0.14)	16.60 (0.72)	16.04 (0.11)	0.56 (0.73)	0.4414
Total SFA (g)	23.07 (0.18)	20.84 (0.75)	23.10 (0.11)	−2.25 (0.75)	**0.0030**
Total sugars (g)	98.64 (0.67)	105.4 (4.77)	98.57 (0.48)	6.86 (4.82)	0.1561
Vitamin A (RE)	785.5 (14.9)	784.2 (56.2)	785.5 (14.7)	−1.30 (55.6)	0.9813
Vitamin B12 (µg)	4.88 (0.11)	3.76 (0.25)	4.89 (0.10)	−1.13 (0.24)	**<0.0001**
Vitamin B6 (mg)	1.85 (0.02)	1.87 (0.08)	1.85 (0.02)	0.03 (0.08)	0.7469
Vitamin C (mg)	89.09 (1.01)	125.6 (8.26)	88.75 (0.98)	36.81 (8.53)	**<0.0001**
Vitamin D (D2 + D3) (µg)	4.73 (0.06)	4.05 (0.37)	4.73 (0.06)	−0.68 (0.37)	0.0650
Vitamin E (ATE) (mg)	7.94 (0.08)	8.42 (0.41)	7.93 (0.07)	0.49 (0.41)	0.2388
Vitamin K ^6^	113.5 (3.95)	121.5 (11.5)	113.4 (3.86)	8.15 (11.7)	0.4859
Zinc (mg)	10.56 (0.11)	9.79 (0.56)	10.57 (0.09)	−0.78 (0.56)	0.1628

Data source: NHANES 1988–1994, 1999–2018; subjects 60 years of age and older (*n* = 18,784) with complete and reliable 24 h dietary recalls. ^1^ Data were adjusted for age, gender, ethnicity, physical activity level, poverty income ratio, weight status, current smoking status, and energy intake (except for energy). ^2^ Mango consumers were defined as subjects who reported raw mango on either day 1 or day 2 24 h recalls. ^3^ Beta is a regression coefficient indicating differences between consumer and non-consumer. ^4^ *p* values are for difference between consumer and non-consumer intake. ^5^ Choline intake was only available for NHANES 2005–2018 cycles. Choline sample sizes: all (*n* = 10,450); consumers (*n* = 177); non-consumers (*n* = 10,273). ^6^ Folate, DFE, total folate, vitamin K were only available for 2003–2018 cycles. Sample sizes: all (*n* = 14,467); consumers (*n* = 210), non-consumers (*n* = 14,257). Bold values designate significant differences at *p* < 0.05. y: years of age; SE: standard error, MUFA: monounsaturated fatty acids; PUFA: polyunsaturated fatty acids; SFA: saturated fatty acids.

**Table 6:** Healthy Eating Index-2020 ^1^ (HEI-2020) of mango consumers ^2^ and non-consumers among older adults (60+ y), NHANES 1988–1994, 1999–2018, gender-combined data.

HEI-2020 Component	All (*n* = 18,784)	Consumers (*n* = 225)	Non-Consumers (*n* = 18,559)	Consumer vs. Non-Consumer	
	Mean (SE)	Mean (SE)	Mean (SE)	Beta ^3^ (SE)	*p* ^4^
Component 1—total vegetables	3.36 (0.02)	3.62 (0.15)	3.36 (0.02)	0.26 (0.15)	0.0891
Component 2—greens and beans	1.47 (0.03)	2.06 (0.26)	1.47 (0.03)	0.59 (0.26)	**0.0236**
Component 3—total fruit	2.67 (0.02)	3.77 (0.16)	2.66 (0.02)	1.11 (0.17)	**<0.0001**
Component 4—whole fruit	2.70 (0.02)	4.06 (0.15)	2.68 (0.02)	1.38 (0.15)	**<0.0001**
Component 5—whole grains	3.12 (0.04)	3.20 (0.46)	3.12 (0.04)	0.07 (0.46)	0.8708
Component 6—dairy	5.09 (0.04)	5.09 (0.33)	5.09 (0.04)	−0.002 (0.33)	0.9958
Component 7—total protein foods	4.19 (0.01)	4.19 (0.13)	4.19 (0.01)	0.003 (0.13)	0.9795
Component 8—seafood and plant protein	2.33 (0.03)	2.74 (0.22)	2.33 (0.03)	0.41 (0.22)	0.0606
Component 9—fatty acid ratio	5.14 (0.05)	6.32 (0.34)	5.13 (0.05)	1.19 (0.35)	**0.0008**
Component 10—sodium	4.15 (0.04)	4.85 (0.37)	4.15 (0.04)	0.71 (0.37)	0.0572
Component 11—refined grain	6.45 (0.04)	6.83 (0.37)	6.45 (0.04)	0.39 (0.37)	0.3009
Component 12—saturated fat	5.97 (0.05)	6.61 (0.34)	5.96 (0.04)	0.65 (0.34)	0.0559
Component 13—added sugar	7.23 (0.04)	7.60 (0.29)	7.23 (0.03)	0.37 (0.29)	0.2065
HEI-2020 total score	53.89 (0.19)	60.94 (1.45)	53.82 (0.17)	7.12 (1.47)	**<0.0001**

Data source: NHANES 1988–1994, 1999–2018; subjects aged 60 years and older (*n* = 18,784) with complete and reliable 24 h dietary recalls. ^1^ Data were adjusted for age, gender, ethnicity, physical activity level, poverty income ratio, weight status, and current smoking status. ^2^ Mango consumers were defined as subjects who reported raw mango on either day 1 or day 2 24 h recalls. ^3^ Beta is a regression coefficient indicating differences between consumer and non-consumer. ^4^ *p* values are for difference between consumer and non-consumer intake. Bold values designate significant differences at *p* < 0.05. y: years of age; SE: standard error.

**Table 7:** Food group intake ^1^ of mango consumers ^2^ and non-consumers among older adults (60+ y), NHANES 1988–1994, 1999–2018, gender-combined data.

Food Group	All (*n* = 18,784)	Consumers (*n* = 225)	Non-Consumers (*n* = 18,559)	Consumer vs. Non-Consumer	
	Mean (SE)	Mean (SE)	Mean (SE)	Beta ^3^ (SE)	*p* ^4^
Beef, poultry, seafood (oz eq)	4.01 (0.04)	3.27 (0.22)	4.01 (0.04)	−0.74 (0.23)	**0.0012**
Refined grain (oz eq)	4.65 (0.04)	4.33 (0.30)	4.65 (0.03)	−0.32 (0.30)	0.2746
Solid fats (g)	32.85 (0.29)	27.95 (1.40)	32.89 (0.23)	−4.94 (1.44)	**0.0007**
Total dairy (cup eq)	1.38 (0.02)	1.33 (0.10)	1.38 (0.01)	−0.05 (0.10)	0.5965
Total fruits (cup eq)	1.10 (0.02)	1.80 (0.12)	1.09 (0.01)	0.71 (0.12)	**<0.0001**
Total grain (oz eq)	5.60 (0.04)	5.45 (0.28)	5.60 (0.03)	−0.15 (0.28)	0.5874
Total mpf, organ, cured, seafood, eggs, soy, nuts, seeds (oz eq)	5.25 (0.05)	4.58 (0.24)	5.25 (0.04)	−0.67 (0.24)	**0.0054**
Total vegetables excluding legumes (cup eq)	1.58 (0.02)	1.76 (0.11)	1.58 (0.01)	0.18 (0.12)	0.1175
Whole grain (oz eq)	0.95 (0.02)	1.12 (0.25)	0.95 (0.02)	0.17 (0.25)	0.4821

Data source: NHANES 1988–1994, 1999–2018; subjects aged 60 years and older (*n* = 18,784) with complete and reliable 24 h dietary recalls. ^1^ Data were adjusted for age, gender, ethnicity, physical activity level, poverty income ratio, weight status, current smoking status, and energy intake. ^2^ Mango consumers were defined as subjects who reported raw mango on either day 1 or day 2 24 h recalls. ^3^ Beta is a regression coefficient indicating differences between consumer and non-consumer. ^4^ *p* values are for difference between consumer and non-consumer intake. Bold values designate significant differences at *p* < 0.05. y: years of age; SE: standard error; mpf: meat and poultry foods.

## Data Availability

The data presented in this study are from publicly available data in NHANES and other additional data are available in the article and Supplementary Materials.

## Supplementary Materials

The following supporting information can be downloaded at: https://www.mdpi.com/article/10.3390/nu16020303/s1, Table S1: Percentage of older adults who follow vegetarian/vegan diets stratified by mango consumer status.
