# Supplementary material for: Mango Consumption Was Associated with Higher Nutrient Intake and Diet Quality in Women of Childbearing Age and Older Adults
Source: Nutrients. 2024 Jan 19;16(2):303. doi: 10.3390/nu16020303 (PMC10820848; doi:10.3390/nu16020303)
Supplement: Supplementary file 1 [file nutrients-16-00303-s001.zip › nutrients-2796107-supplementary.pdf]

Supplementary Table S1. Percentage of older adults who follow vegetarian/vegan diets stratified by mango consumer status[1,2]

Gender	All		Consumers		Non-Consumers		Consumer vs Non-Consumer	
	N	Mean (SE)	N	Mean (SE)	N	Mean (SE)	Beta[3] (SE)	P[4]
**Self Identified as Vegetarian**								
All	3,399	1.61 (0.25)	35	3.87 (2.90)	3,364	1.59 (0.26)	2.28 (2.96)	0.4467
Male	1,694	1.08 (0.23)	13	1.98 (2.82)	1,681	1.08 (0.23)	0.90 (2.79)	0.7496
Female	1,705	2.04 (0.42)	22	5.11 (3.84)	1,683	2.00 (0.43)	3.11 (3.98)	0.4407
**Subjects with Zero Meat Intake**								
All	18,784	6.91 (0.29)	225	15.36 (4.29)	18,559	6.83 (0.29)	8.53 (4.31)	**0.0493**
Male	9,371	5.29 (0.36)	98	8.66 (4.34)	9,273	5.27 (0.37)	3.39 (4.40)	0.4418
Female	9,413	8.23 (0.41)	127	20.24 (5.54)	9,286	8.11 (0.40)	12.14 (5.54)	**0.0297**

Data source: NHANES 1988-1994, 1999-2018; subjects age 60 years of age and older (n=18,784) with complete and reliable 24-hr dietary recalls. [1]Data were adjusted for age, gender, ethnicity, physical activity level, poverty income ratio, weight status, and current smoking status. [2]Mango consumers were defined as subjects who reported raw mango on either day 1 or day 2 24-hour recalls. [3]Beta is a regression coefficient indicating differences between consumer and non-consumer. [4]p values are for difference between consumer and non-consumer intake. Bold values designate significant differences at p<0.05. y: years of age; SE: standard error.
